# Platelet activation in patients with peripheral artery disease from 43 sites: Assessment of samples transported by overnight mail

**DOI:** 10.1016/j.jvsvi.2025.100197

**Published:** 2025-03-22

**Authors:** William B. Stubblefield, Alexander E. Sullivan, Katherine N. Cahill, Taneem Amin, Olivier Boutaud, Irene Zagol-Ikapitte, Yan Ru Su, David K. Flaherty, Brittany K. Matlock, Jeffrey Berger, Joshua A. Beckman

**Affiliations:** a Department of Emergency Medicine, Vanderbilt University Medical Center;; b Department of Cardiovascular Medicine, Vanderbilt University Medical Center;; c Division of Allergy, Pulmonary, and Critical Care, Department of Medicine, Vanderbilt University Medical Center;; d Warren Center for Neuroscience Drug Discovery, Department of Pharmacology, Vanderbilt University;; e Flow Cytometry Shared Resource, Vanderbilt University Medical Center;; f Department of Medicine, New York University Langone Health;; g Division of Vascular Medicine, Department of Medicine, University of Texas Southwestern Medical Center.

**Keywords:** Platelets, PAR-1, Platelet activation, Specimen transport, Peripheral artery disease

## Abstract

**Background:**

Platelet activation plays a key role in peripheral artery disease, but the relevant platelet activation mechanisms in chronic limb-threatening ischemia remain unclear. Moreover, multicenter studies of in vivo platelet activation remain difficult owing to methodological limitations. We report the feasibility of a novel method to measure in vivo platelet activation using samples sent via overnight mail. We hypothesized that platelet function would be measured reliably after overnight transport.

**Methods:**

Peripheral venous blood was drawn from 190 participants with severe peripheral artery disease at 43 sites in BEST-CLI (Best Endovascular vs Best Surgical Therapy in Patients with Chronic Limb Ischemia) and shipped by Federal Express cold pack from study site locations within the continental United States to Vanderbilt University Medical Center from July 2017 to November 2019. Fifty-six volunteers with and without diabetes were recruited locally as time- and age-matched control subjects. Samples were collected using a platelet activation inhibitor. Markers of platelet activation, thromboxane B2, dilysyl-malondialdehyde (MDA), p-selectin, WEDE 15, and SPAN 12 were measured using liquid chromatography with tandem mass spectrometry and flow cytometry. The primary objective was to report baseline markers of platelet activation from samples sent by overnight mail compared with an age-matched population recruited locally without known atherosclerosis.

**Results:**

Compared with control subjects, patients from BEST-CLI were more likely to be male (72.6% vs 51.8%; *P* < .01), have prior atherosclerotic cardiovascular disease (22.1% vs 0%; *P* < .01), and be prescribed aspirin (69.4% vs 35.7%; *P* < .01) or clopidogrel (20.0% vs 0%; *P* < .01). Thromboxane B2 (0.5 vs 2.9 ng/mL; *P* < .01) and dilysyl-MDA (4.7 vs 9.7 ng/mg; *P* < .01) were significantly higher in BEST-CLI patients than control subjects and p-selectin (592.0 meaajpan fluorescent intensity vs 420.5 mean fluorescent intensity; *P* < .01) was lower. Levels of thromboxane B2 and dilysyl-MDA varied significantly and as expected across the range of antithrombotic treatment intensity (*P* < .01).

**Conclusions:**

Measurement of platelet activation after sample shipment on cold pack is feasible and yields reliable results that correspond with locally obtained clinical values.

Peripheral artery disease (PAD) is a form of atherosclerosis in the arteries of the lower extremities that affects 10 million Americans and more than 200 million persons globally.^1,2^ As a cardinal manifestation of systemic atherosclerosis, PAD is associated with an increased risk of myocardial infarction, stroke, and death.^3^ Moreover, PAD impairs lower limb function such that patients may present for medical attention for intermittent claudication or chronic limb-threatening ischemia (CLTI).^4^ The latter is associated with a high rate of amputation in the absence of revascularization and wound care.

BEST-CLI (Best Endovascular vs Best Surgical Therapy in Patients With Chronic Limb Ischemia) is a randomized controlled treatment strategy trial that investigated first use of surgical bypass compared with endovascular revascularization (NCT02060630).^5^ The use of medical therapy in patients in BEST-CLI was modest, with 73% of patients at study entry taking an antiplatelet agent.^6^ Antiplatelet therapy is a cornerstone of PAD care to reduce both cardiac and limb events.^4^

The impact of antiplatelet therapy on lower limb outcomes remains unexceptional when studying cyclo-oxygenase inhibition with aspirin and adenosine diphosphate inhibition with P2y12 inhibitors.^7^ In contrast, there is evidence that inhibition of protease-activated receptor-1 (PAR-1), the putative thrombin receptor, reduces adverse lower limb events in PAD.^8,9^ TIDE (The Impact of Diabetes on Revascularization), a substudy of BEST-CLI (NCT03085524), recruited subjects from 43 sites to understand the role of PAR-1 activation (as measured by WEDE 15 and SPAN 12, antibodies that recognize epitopes on the thrombin receptor) in CLTI.^10^ Here we report baseline markers of platelet activation from patients with TIDE samples sent by overnight mail compared with an age-matched population recruited locally without known atherosclerosis using previously established methods.^11^ We hypothesized that platelet function would be in the expected range after overnight transport and that platelet function would be modulated by antiplatelet use as seen in other real-world reports.

## METHODS

### Design and setting.

This was a substudy (TIDE; NCT03085524) within BEST-CLI to investigate mechanisms of revascularization failure among a subgroup of participants. BEST-CLI was an investigator-initiated, international, multispecialty, prospective, randomized, open-label, pragmatic superiority trial designed to assess the efficacy of a surgery-first vs an endovascular-first revascularization strategy in patients with CLTI who were candidates for both revascularization strategies.^5^ At a subset of study sites, participants for whom duplex ultrasound surveillance imaging of the index intervention was planned were recruited in parallel with BEST-CLI from July 2017 to November 2019. All components of the substudy were synchronous with the BEST-CLI trial, with laboratory and imaging assessment collected by the trial team in parallel with the BEST-CLI trial protocol. Because all substudy procedures were conducted under the umbrella of the parent study and subject data were deidentified to the TIDE study team, this substudy was institutional review board exempt. All locally recruited participants provided written, informed consent.

### Blood sample collection.

Three 3-mL Blue top BD Vacutainer Sodium Citrate tube (BD Cat# 363,083; Franklin Lakes, NJ) were collected. Samples were combined with the platelet inhibitor and were shipped FedEx priority overnight in a Therapak Insulated shipper with cold pack. Upon receipt, samples were immediately stored at −20°C until use. One 10-mL BD Vacutainer EDTA tube (BD Cat#366643), and one 10-mL BD Vacutainer tube (BD Cat#367820), were used for plasma and serum collection, respectively.

### Data collection.

Demographic and clinical data, including medical history and medications, were collected by the BEST-CLI study team in accordance with the study protocol. All data were defined in an identical manner to that described in BEST-CLI.^5^

### Sample analysis.

**Fig 1.**
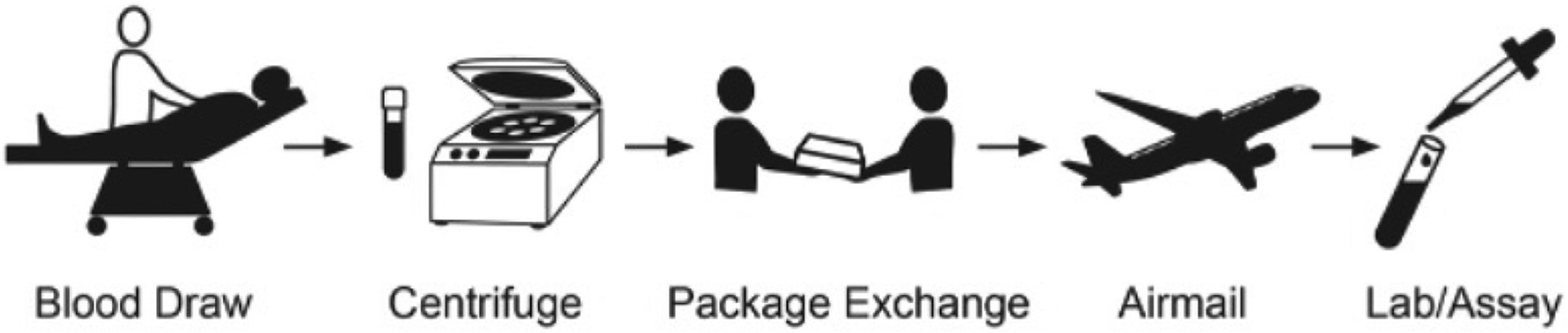
Novel assay methodology involving platelet transport and analysis.

Whole blood, serum, and plasma samples were collected by the study team on enrollment into the parent trial. A platelet-stabilizing agent (Patent No. 10,166,248 and 10,688,109), previously demonstrated to freeze the platelet activation state at the time of collection and inhibit further in vitro platelet activation, was added to whole blood samples to permit delayed assessment.^12^ Samples were then shipped to Vanderbilt University Medical Center (VUMC) on cold pack overnight by FedEx. Once the samples were received at VUMC, platelet activation markers, CD41a-APC (BD #559777), p-selectin (CD62P-BV421- BD #564038), Anti-Thrombin Receptors WEDE 15 (Beckman Coulter #IM2584; Brea, CA) and SPAN 12 (Beckman Coulter #IM2583), and PAC-11 FITC (BD #340507) were analyzed by flow cytometry using whole blood collected in the sodium citrate tube with the platelet inhibitors on the following day. Preparation of the platelet stabilizing reagent has been previously described and is composed of aspirin (Sigma #A5376; St. Louis, MO), apyrase (Sigma #A6635), carbaprostacyclin (Cayman Chemical #18210; Ann Arbor, MI), and SQ 29,548 (Cayman Chemical #19025).^11^ PBS was obtained from Gibco (Thermo Fisher Scientific #10010–023; Waltham, MA). The dilysyl-malondialdehyde (MDA) crosslink’s analysis was reported previously.^11,13^ Analyst software version 1.7.3 (Sciex, Toronto, Ontario, Canada) was used for data acquisition, and samples were quantified using the companion software MultiQuant 3.0 (Sciex). Quantification of the dilysyl-MDA crosslinks in samples was performed using the standard curve and a weighted least-squares regression analysis (1/×2) by measuring the peak area ratio of the analytes to the internal standard. Serum thromboxane B2 levels were assessed by stable isotope dilution gas chromatography/mass spectrometry with selective ion monitoring (Fig 1).

### End points.

The study goal was to determine the feasibility of platelet function testing after overnight shipping. The primary endpoint of interest was whether platelet function in our study was in the expected range based on prior cohorts. Secondary exploratory end points included the effect of diabetes, aspirin, and P2Y12 inhibitors on measures of platelet function.

### Statistical analysis.

Continuous variables were reported as median values with interquartile ranges (IQRs). Between group comparisons were conducted using Wilcoxon rank-sum test. Categorical variables are reported as total number (%) with comparisons made using Pearson χ^2^ test or Fisher’s exact test as appropriate. Outcomes of interest were markers of platelet activity according to collection location (controls on site vs patients with CLTI at recruitment centers). An interaction effect between aspirin use and diabetes was assessed using a two-way analysis of variance test. Data were analyzed using R version 4.2.2 for Mac (The R Foundation for Statistical Computing, Vienna, Austria).

## RESULTS

### Clinical characteristics.

Clinical characteristics of the study cohort are shown in Table I. A total of 246 patients, including 56 control participants and 190 patients with CLTI were included. The median age was 67.0 years (IQR, 63.0–73.3 years), and patients included 79 (32.1%) females and 31 (12.6%) Blacks. Patients with CLTI were significantly more likely to be male (72.6% vs 51.8%; *P* = .006), have a smoking history (83.1% vs 25.0%; *P* < .001), and have had prior myocardial infarction (22.1% vs 0.0%; *P* < .001). They were also significantly more likely to be on aspirin (68.4% vs 35.7%; *P* < .001) and clopidogrel (20.0% vs 0.0%; *P* < .001).

### Markers of platelet function and PAR-1 activation.

Platelet function and PAR-1 activation results are shown in Table II. Patients with CLTI had significantly lower levels of thromboxane B2 (median, 0.48 ng/mL [IQR, 0.00–2.24 ng/mL] vs 2.90 ng/mL [IQR, 0.00–6.69 ng/mL]; *P* = .003) and dilysyl-MDA (4.72 ng/mg [IQR, 2.71–6.90 ng/mg] vs 9.75 ng/mg [IQR, 7.42–12.18 ng/mg]; *P* < .001), and higher levels of CD62 (592.00 mean fluorescent intensity [MFI] [IQR, 472.00–748.50 MFI] vs 420.50 MFI [IQR, 273.50–731.88 MFI]; *P* = .001). Markers of PAR-1 activation, including WEDE 15 = (806.50 MFI [IQR, 693.25–973.25 MFI] vs 831.50 MFI [IQR, 722.50–881.00 MFI]; *P* = .918) and SPAN 12 (612.26 MFI [IQR, 542.94–711.03 MFI] vs 609.88 MFI [IQR, 547.99–658.96 MFI]; *P* = .472), were similar between the two groups.

### Effect of diabetes and aspirin use on platelet function and PAR-1 activation.

There was no significant difference in any marker of platelet activation between those with and without diabetes (Table III). In those patients on aspirin therapy, thromboxane B2 (0.33 ng/mL [IQR, 0.00–1.17 ng/mL] vs 2.73 ng/mL [IQR, 0.32–6.44 ng/mL]; *P* < .001) and dilysyl-MDA (5.01 ng/mg [IQR, 2.96–7.96 ng/mg] vs 7.68 ng/mg [IQR, 4.72–11.00 ng/mg]; *P* = .002) were significantly lower than in those not on aspirin = (Table III). Levels of CD62, WEDE 15, and SPAN 12 were similar between those with and without aspirin use. There was no significant interaction between diabetes status and aspirin use for any measure of platelet function or PAR-1 activation.

### Effect of antiplatelet regimen on platelet function and PAR-1 activation.

**Fig 2.**
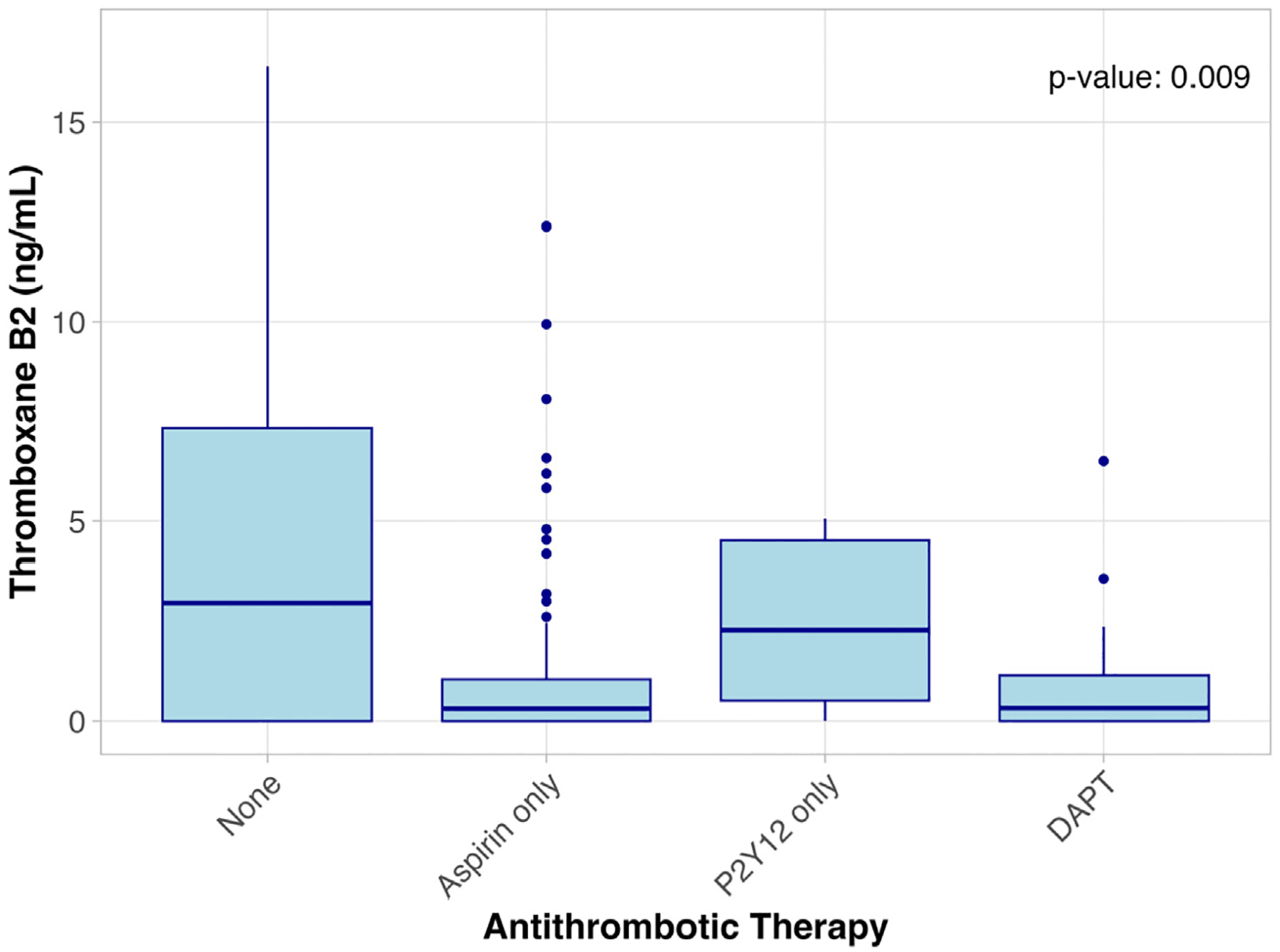
Thromboxane B2 levels in patients taking antithrombotic agents. *DAPT*, dual antiplatelet therapy.

**Fig 3.**
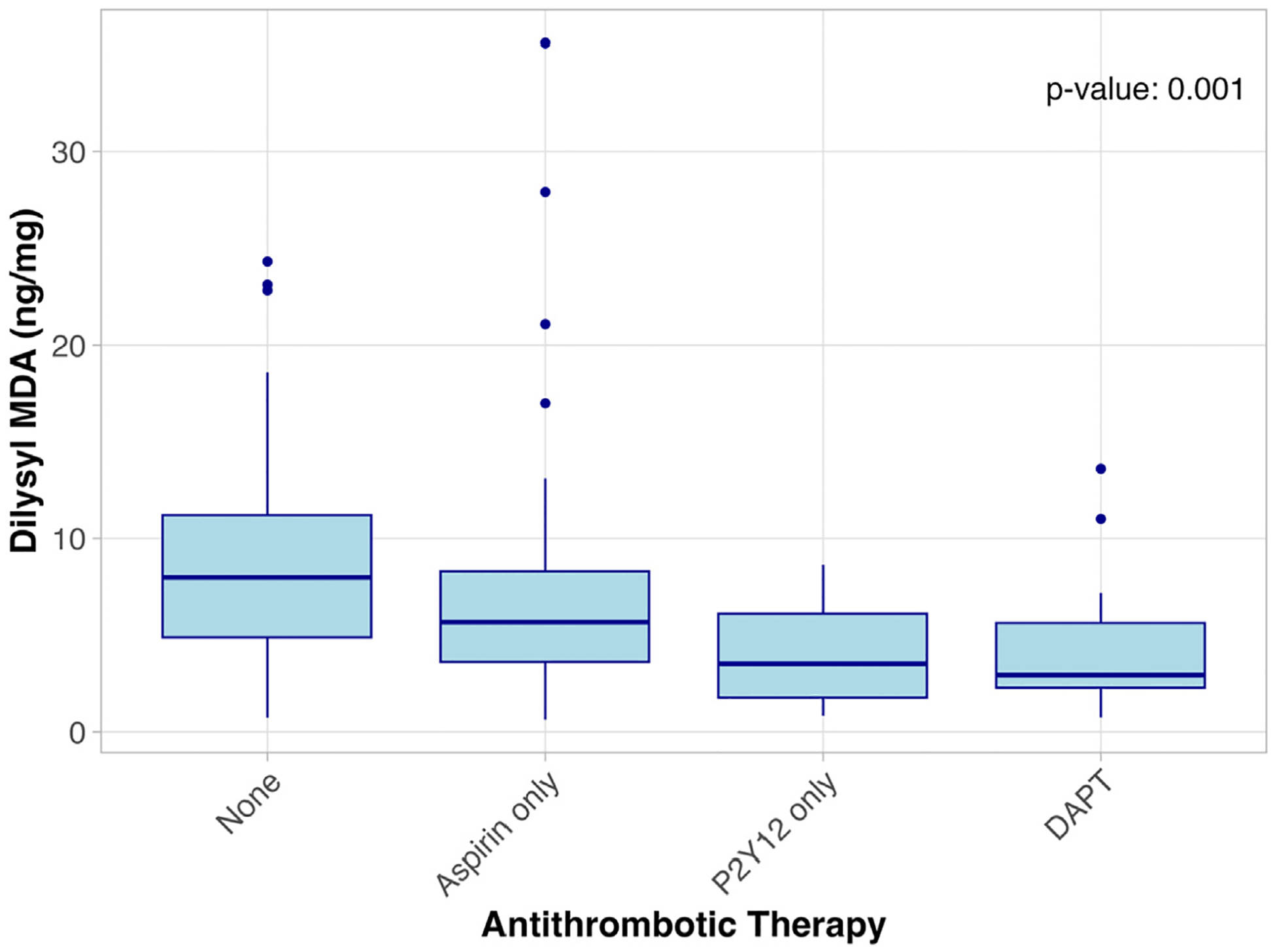
Dilysyl malondialdehyde (MDA) levels in patients taking antithrombotic agents. *DAPT*, dual antiplatelet therapy.

Across all study participants, 79 (34.5%) were on no antiplatelet agents, 109 (47.6%) were on aspirin only, 10 (4.4%) were on only a P2Y12 agent, and 31 (13.5%) were on dual antiplatelet therapy (DAPT). Thromboxane B2 levels were significantly lower among those on aspirin alone or DAPT compared with no antiplatelet agents or P2Y12 alone (*P* = .009) (Table IV and Fig 2). Dilysyl-MDA levels progressively declined as the intensity of antiplatelet therapy increased from no therapy to DAPT (*P* = .001) (Fig 3).

## DISCUSSION

In this feasibility study, we report markers of platelet function and PAR-1 activation among patients with CLTI and controls after overnight shipment of blood samples on cold packs. Patients with CLTI exhibited lower concentrations of thromboxane B2, dilysyl-MDA, and CD62 compared with controls, a finding consistent with the differential use of antiplatelet agents, whereas levels of WEDE 15 and SPAN 12 remained similar. This observation was further substantiated after demonstrating lower thromboxane B2 levels in those on aspirin-containing regimens and lower dilysyl-MDA concentrations as antiplatelet regimen intensified. Importantly, all markers of platelet activity were within the expected range based on previous reports, affording proof-of-concept evidence for this method.^11,14–16^

Platelet activation is a critical event after plaque destabilization that contributes to cardiovascular events.^17^ After endothelial disruption, platelets interact with the underlying vascular collagen fibrils, which provide a scaffold for adhesion and a strong signal for platelet activation. Thromboxane and adenosine diphosphate are secreted and activate neighboring platelets while fibrin allows for crosslinks between receptors on activated platelets. Thromboxane, P-selectin, and thrombin-induced PAR-1 activation are key mediators of this process, and overactivation of these targets has been linked to increased risk of cardiovascular events across the spectrum of cardiovascular disease.^18–20^

In contrast with coronary artery disease, an effective antithrombotic regimen in patients with PAD was elusive for many years. Aspirin was widely prescribed to this patient population with little evidence suggesting a reduction in limb vascular events.^21^ Although randomized trials showed a decrease in major adverse cardiovascular events with clopidogrel and ticagrelor, there was no difference in vascular outcomes, even when P2Y12 inhibitors were co-administered with aspirin.^7,22–24^ Vorapaxar, a PAR-1 antagonist, was the first agent to demonstrate a reduction in limb events in patients with symptomatic PAD, largely driven by a reduction in surgical graft thrombosis.^8^ Low-dose rivaroxaban, similarly, was shown to decrease major limb events in patients with symptomatic PAD and after revascularization when combined with aspirin.^25,26^ Although the anticoagulant effects of factor Xa inhibition are well-established, rivaroxaban also exhibits an antiplatelet effect by reducing factor Xa agonism of PAR-1.^27^ These data suggest that PAR-1 inhibition is a key therapeutic target and that the assessment of platelet and PAR-1 activation may be a means to personalize antithrombotic therapy.

To better understand the role of platelet activation in patients with CLTI, we used established methods with the addition of a platelet stabilization agent to facilitate overnight shipment on cold packs.^11,12^ Current inhibitors of platelet activation used during routine phlebotomy only prevent platelet activation for 2 hours and are therefore insufficient for overnight shipping.^11^ Typically, PGH2 is metabolized by thromboxane synthase to equal amounts of MDA and thromboxane A2. MDA subsequently forms adducts of proteins, creating the substrate for subsequent intra and intermolecular cross-links. The addition of a scavenger compound preferentially reacts with MDA and forms a stable nonreactive adduct, halting the process of platelet activation and facilitating overnight shipping.^11^ Our validation of these methods has important implications as we begin to understand the prognostic value of these platelet markers in large-scale randomized trials such as BEST-CLI. Although not every laboratory will have the infrastructure for platelet function testing, these methods make it feasible to have centralized core labs to make platelet function testing available as a send out test, for both investigational and clinical possibilities, to understand the correlation with adverse limb and cardiovascular outcomes.

Prior studies using on-site platelet function testing have demonstrated a significant, dose response reduction in both thromboxane B2 and dilysyl-MDA level with aspirin therapy via cyclo-oxygenase-2 (COX-2)-dependent pathways.^11,15,16^ We demonstrate a similar pattern in our cohort even after overnight shipment. Direct PAR-1 activation, however, is unaffected by COX-2 inhibition with aspirin and should not be influenced by antiplatelet therapy. As expected, we observed no change in WEDE 15 or SPAN 12 levels in those treated with aspirin.^28^ Preservation of the relationship between platelet activation and COX-2 inhibition following overnight sample transport provides further evidence for the feasibility and reliability of this methods.

This study, designed to demonstrate feasibility of a novel method of platelet activation assessment, had three notable limitations. First, no patients in our study were prescribed vorapaxar, so we were unable to assess preservation of the relationship between WEDE 15 and SPAN 12 and targeted drug therapy. Second, we did not attempt multivariable adjustment because our goal was not to control for the known differences between the control and CLTI groups, but rather to use these differences to ensure established patterns of platelet function with drugs and comorbidities were preserved. Third, we report results from a subset of the parent BEST-CLI trial. Given the limited sample size, there may be unaccounted selection bias; however, this factor should not impact the results of platelet function testing. Given the validity and reliability of this novel platelet transportation method, this method may be suitable to explore the relationship between platelet activation and rate of limb and cardiovascular events across a range of participating sites.

## CONCLUSIONS

We demonstrate the feasibility of overnight shipment of samples on cold packs for assessment of platelet function. Using this method, we found that measures of thromboxane B2, dilysyl-MDA, CD62, WEDE 15, and SPAN 12 are within expected ranges and the effects of antiplatelet therapy on platelet reactivity are preserved. Future work will associate markers of platelet reactivity with limb and cardiovascular outcomes.

Authors acknowledge the Vanderbilt Cardiology Core Lab for their contributions coordinating the platelet function assay and providing the sample collection kit.

## Figures and Tables

**Table I.** Baseline characteristics between patients with and without chronic limb-threatening ischemia (*CLTI*)

	CLTI (n = 190)	Control (n = 56)	*P* value
Age,	67.3 [61.8–74.3]	66.5 [63.0–70.0]	.696
Female sex	52 (27.4)	27 (48.2)	.006
Race			
White	147 (77.4)	50 (89.3)	.135
Black	27 (14.2)	4 (7.1)	
Hispanic	7 (3.7)	0 (0.0)	
Native American or Alaskan Native	6 (3.2)	0 (0.0)	
Asian	2 (1.1)	2 (3.6)	
Other	1 (0.5)	0 (0.0)	
BMI (kg/m^2^)	27.3 [23.5–32.1]	29.3 [26.9–33.5]	.014
Diabetes mellitus	124 (65.3)	29 (51.8)	.095
Hypertension	163 (85.8)	34 (60.7)	<.001
Hyperlipidemia	139 (73.2)	41 (73.2)	.999
Prior MI	42 (22.1)	0 (0.0)	<.001
Smoking history			
Current	85 (44.7)	0 (0.0)	<.001
Former	73 (38.4)	14 (25.0)	
Never	32 (16.8)	42 (75.0)	
Creatinine, mg/dL^a^	1.0 [0.8–1.3]	0.9 [0.8–1.0]	.002
Medications			
Warfarin	7 (3.7)	0 (0.0)	.357
Heparin	32 (16.8)	0 (0.0)	<.001
Lovenox	3 (1.6)	0 (0.0)	.999
Apixaban	10 (5.3)	1 (1.8)	.464
Rivaroxaban	4 (2.1)	0 (0.0)	.576
Aspirin	130 (68.4)	20 (35.7)	<.001
Clopidogrel	38 (20.0)	0 (0.0)	<.001
Ticagrelor	3 (1.6)	0 (0.0)	.999
Prasugrel	2 (1.1)	0 (0.0)	.999
Statin	142 (74.7)	34 (60.7)	.069
ACEi or ARB	93 (48.9)	29 (51.8)	.709

*ACEi*, Angiotensin-converting enzyme inhibitor; *ARB*, angiotensin receptor blocker; *BMI*, body mass index; *MI*, myocardial infarction.

Values are median [interquartile range] or number (%).

*P* values calculated using Mann-Whitney *U* tests for continuous variables and Pearson χ^2^ or Fisher’s Exact tests (as appropriate) for categorical variables.

Two patients with CLTI were missing data on medications.

Five patients with CLTI were missing creatinine values.

a Renal function for CTLI vs control, n (%), respectively: normal renal function (glomerular filtration rate ≥60 mL/min/1.732 m^2^) = 153 (80.5) and 56 (100.0); stage 3 (glomerular filtration rate 30–59 mL/min/1.732 m^2^) = 17 (8.9) and 0 (0.0); stage 4 (glomerular filtration rate 15–29 mL/min/1.732 m^2^) = 2 (1.1) and 0 (0.0); stage 5 (glomerular filtration rate <15 mL/min/1.732 m^2^) = 1 (0.5) and 0 (0.00); dialysis dependent (end-stage renal disease) = 13 (6.8) and 0 (0.0); functioning renal transplant = 4 (2.1) and 0 (0.0).

**Table II.** Markers of platelet activation in patients with chronic limb-threatening ischemia (CLTI) and controls

	CLTI (n = 190)	Control (n = 56)	*P* value
Thromboxane B2 (ng/mL)	0.48 [0.00–2.24]	2.90 [0.00–6.69]	.003
Dilysyl-MDA (ng/mg)	4.72 [2.71–6.90]	9.75 [7.42–12.18]	<.001
CD62 (P-selectin) (MFI)	592.00 [472.00–748.50]	420.50 [273.50–731.88]	.001
WEDE 15 (MFI)	806.50 [693.25–973.25]	831.50 [722.50–881.00]	.918
SPAN 12 (MFI)	612.26 [542.94–711.03]	609.88 [547.99–658.96]	.472

*CLTI*, Chronic limb-threating ischemia; *MDA*, malondialdehyde; *MFI*, mean fluorescent intensity.

Values are median [interquartile range].

*P* values calculated using Mann-Whitney *U* tests.

**Table III.** Markers of platelet activation in patients with and without diabetes mellitus and aspirin use

	No diabetes (n = 93)	Diabetes (n = 153)	*P* value	No aspirin use (n = 94)	Aspirin use (n = 150)	*P* value	*P* value for interaction
Thromboxane B2 (ng/mL)	1.14 [0.00–4.75]	0.48 [0.00–2.54]	.104	2.73 [0.32–6.44]	0.33 [0.00–1.17]	<.001	.215
Dilysyl-MDA (ng/mg)	6.88 [4.26–10.05]	5.58 [3.27–8.62]	.223	7.68 [4.72–11.00]	5.01 [2.96–7.96]	.002	.300
CD62 (P-selectin) (MFI)	528.50 [414.38–686.75]	590.50 [454.50–764.75]	0.363	544.50 [397.25–746.62]	584.75 [454.88–734.62]	.299	.538
WEDE 15 (MFI)	818.00 [710.50–972.25]	815.50 [695.50–968.00]	.914	831.00 [709.25–946.00]	813.50 [701.50–970.50]	.636	.451
SPAN 12 (MFI)	604.52 [544.72–686.04]	612.85 [542.94–691.09]	.855	596.19 [536.10–664.32]	617.61 [553.05–701.80]	.139	.439

*MDA*, Malondialdehyde; *MFI*, mean fluorescent intensity.

Values are median [interquartile range].

*P* values for between group comparisons (by diabetes status and aspirin use) were calculated using Mann-Whitney *U* tests. Interaction between diabetes and aspirin use was tested using a two-way analysis of variance.

**Table IV.** Markers of Platelet Function in Patients taking Antiplatelet Agents^a^

	None (n = 79)	Aspirin only (n = 109)	P2Y12 only (n = 10)	DAPT (n = 31)	*P* value
Thromboxane B2 (ng/mL)	2.96 [0–7.33]	0.313 [0–1.05]	2.28 [0.516–4.52]	0.326 [0–1.14]	.009
Dilysyl-MDA (ng/mg)	7.97 [4.91–11.2]	5.70 [3.64–8.28]	3.54 [1.78–6.14]	2.96 [2.31–5.66]	.001
CD62 (P-selectin) (MFI)	551 [397–730]	579 [442–754]	539 [439–891]	605 [513–651]	.853
WEDE 15 (MFI)	830 [718–928]	827 [664–979]	889 [665–1220]	779 [713–922]	.919
SPAN 12 (MFI)	594 [540–649]	615 [549–686]	605 [544–807]	660 [597–768]	.312

*DAPT*, Dual antiplatelet therapy; *DOAC*, direct oral anticoagulant; *MDA*, malondialdehyde; *MFI*, mean fluorescent intensity.

Values are median [interquartile range].

*P* values generated with analysis of variance tests.

a Mutually exclusive groups.

## Data Availability

The data that support the findings of this study are available from the corresponding author, JAB, upon reasonable request.

## ARTICLE HIGHLIGHTS

- **Type of Research:** Multicenter prospective cohort study
- **Key Findings:** Using 190 venous blood samples from the Best Endovascular vs Best Surgical Therapy in Patients with Chronic Limb Ischemia trial and 56 control participants, we demonstrate feasibility of platelet function testing after overnight shipping on cold packs.
- **Take Home Message:** Off-site platelet function testing is feasible and may permit widespread use of testing for investigational and clinical implications.
